# The right to health of non-nationals and displaced persons in the sustainable development goals era: challenges for equity in universal health care

**DOI:** 10.1186/s12939-016-0500-z

**Published:** 2017-02-21

**Authors:** Claire E. Brolan, Lisa Forman, Stéphanie Dagron, Rachel Hammonds, Attiya Waris, Lyla Latif, Ana Lorena Ruano

**Affiliations:** 1 School of Public Health, University of Queensland, Queensland, Australia; 2 Dalla Lana School of Public Health, University of Toronto, Toronto, Canada; 3 Global Studies Institute/Faculty of Law, University of Geneva, Geneva, Switzerland; 4 Law and Development Research Group, Faculty of Law, University of Antwerp, Antwerp, Belgium; 5 University of Nairobi, Nairobi, Kenya; 6 Center for the Study of Governance and Equity in Health Systems Guatemala City, Guatemala, Guatemala; 7 Center for International Health, University of Bergen, Bergen, Norway

**Keywords:** Sustainable development goals, Millennium development goals, Refugees, Health of non-nationals, Right to health

## Abstract

**Introduction:**

Under the Millennium Development Goals (MDGs), United Nations (UN) Member States reported progress on the targets toward their general citizenry. This focus repeatedly excluded marginalized ethnic and linguistic minorities, including people of refugee backgrounds and other vulnerable non-nationals that resided within a States’ borders. The Sustainable Development Goals (SDGs) aim to be truly transformative by being made operational in all countries, and applied to all, nationals and non-nationals alike. Global migration and its diffuse impact has intensified due to escalating conflicts and the growing violence in war-torn Syria, as well as in many countries in Africa and in Central America. This massive migration and the thousands of refugees crossing borders in search for safety led to the creation of two-tiered, ad hoc, refugee health care systems that have added to the sidelining of non-nationals in MDG-reporting frameworks.

**Conclusion:**

We have identified four ways to promote the protection of vulnerable non-nationals’ health and well being in States’ application of the post-2015 SDG framework: In setting their own post-2015 indicators the UN Member States should explicitly identify vulnerable migrants, refugees, displaced persons and other marginalized groups in the content of such indicators. Our second recommendation is that statisticians from different agencies, including the World Health Organization’s Gender, Equity and Human Rights programme should be actively involved in the formulation of SDG indicators at both the global and country level. In addition, communities, civil society and health justice advocates should also vigorously engage in country’s formulation of post-2015 indicators. Finally, we advocate that the inclusion of non-nationals be anchored in the international human right to health, which in turn requires appropriate financing allocations as well as robust monitoring and evaluation processes that can hold technocratic decision-makers accountable for progress.

## Background

Under the Millennium Development Goals (MDGs), United Nations (UN) Member States reported progress on the targets toward their general citizenry. This focus repeatedly excluded marginalized ethnic and linguistic minorities, including people of refugee backgrounds and other vulnerable non-nationals that resided within a States’ borders [1, 2]. With multilateral and bilateral impetus on scaling-up MDG achievement in low-income countries, the health inequities experienced by vulnerable and poor minoritiesin middle and high-income nations were further overlooked, this includes non-nationals [3]. Recognizing this inequity, in October 2013 the Goals for Governance and Global Health (Go4Health) research consortium called for the post-2015 Sustainable Development Goals (SDGs) agenda to be truly transformative by being made operational in all countries, and applied to all, nationals and non-nationals alike [4]. Go4Health explicitly asked governments to begin progressively meeting their minimum core right to health obligations for vulnerable populations, such as non-nationals, displaced persons and minorities that live within their borders, and to be responsive to in-country health challenges and inequities resulting from cross-border human movement.

Global migration and its diffuse impact have intensified due to escalating conflicts and growing violence in war-torn countries such as Syria, as well as in many countries in Africa and in Central America. The mass migration of thousands of refugees crossing borders in search for safety has led to many countries creating two-tiered, ad hoc, refugee health care systems that have contributed to the sidelining of non-nationals in MDG-reporting frameworks [2]. This situation underlines the importance of including vulnerable non-nationals in global development frameworks like the SDGs. Thousands of minors from the northern triangle of Central America have made the perilous crossing into the United States, and one million refugees crossed the Mediterranean to enter Europe in 2015. The United Nations High Commissioner for Refugees (UNHCR) reported worldwide displacement had reached the highest ever-recorded level, with 59.5 million people now of concern [5–7]. This is not a problem of ‘the west’, that given Turkey, Pakistan, Lebanon, Iran, Ethiopia and Jordan each host between 1.59 million and 654,100 displaced individuals [5, 6]. Of the 59.5 million people displaced in 2015, approximately one-third (19.5 million) were refugees, the bulk of whom (86%) reside in developing regions, with least developed countries hosting 25% of that total [5, 6].

Tens of millions more still lie outside UNHCR’s mandate, and they are not counted in the 59.5 million figure; these are the undocumented or irregular migrants who have crossed borders for equally complex reasons. These human beings are trying to escape from poverty, from environmental and climate instability, rapid urbanization, human trafficking, slavery, and unimaginable levels of violence [8, 9]. According to UNHCR, the fifteen new or ongoing conflicts throughout the world in the past 5 years only compound these factors. . The Syrian crisis highlights the complex interconnection between war, refugee status and the undocumented movement of people, and is responsible for a large proportion of this burden [5]. Both the refugees escaping from Syria, as well as the minors from Central America are easy prey for international criminal gangs, who engage in human smuggling and trafficking, as well as multiple forms of exploitation of these vulnerable populations [7]. Often those on the move are people in extremely vulnerable positions and are routinely denied the means to take control of their health as well as of their life circumstances [10]. Improving the human health and dignity of these and other groups is and will be a transnational challenge that can only be addressed through global action, solidarity, recognition and commitment by the global community to realize the post-2015 SDG catchcry of ‘leaving no one behind’ [11, 12].

### The health challenges of people on the move

It is important to note that each person that is traversing the world’s borders in challenging circumstances differs in health status and in need. Studies usually focus on healthcare issues and barriers for people in refugee camps or those seeking asylum in high-income nations. Studies on refugee camps in resource-poor settings frequently describe overcrowding, poor hygiene and sanitation, poor water quality, food insecurity, discrimination, and issues of violence (including sexual violence). As a result, camp inhabitants experience multimorbidities, which may include communicable diseases and parasitic infections, under-management of chronic health conditions, under-immunization, and inadequate nutrition. Al of this can result in delayed growth and development in children, as well as sexual and reproductive health problems, among many other conditions. Meanwhile, the literature on refugee health in high-income settings routinely identifies the need for on-arrival health-care screening, as well as identifies the vulnerability of adults and children, particularly unaccompanied minors, to develop mental health problems. Often, a government’s denial of health services and basic social determinants of health is tied to promotion of state securitization and inter-related policies regulating cross-border movements of people [13], but little consideration seems to be given to the migrants and refugees themselves.

### What do the sustainable development goals offer?

It is worth emphasizing that the SDGs, which consist of 17 goals and 169 associated targets, is intended as a universal agenda for all people in all segments of society, in developed and developing countries alike [11]. This means that vulnerable groups such as refugees, internally displaced persons and migrants deserve not only consideration but also health systems that are responsive to their specific health needs. In the post-2015 UN resolution governments have collectively pledged that the SDGs are to include people whose backgrounds are marred by persecution, poverty, extremism, conflict, violence, humanitarian crises, natural disaster, and forced displacement. However such affirmative statements, and those on access to education and humane treatment of persons regardless of migration status, are laudable but not enough.

Governments will likely shape their SDG policies and programs to align with their post-2015 commitments in the SDG metrics framework instead of the commitments found in the UN resolution’s preamble or broader declaration. This reaffirms the growing concern as to the real possibility that the most poor, socially isolated, and disadvantaged groups the SDGs seek to capture will be overlooked and ignored by a multitude of countries and their development partners when it comes to planning, implementation, monitoring, and reporting within the SDG framework. These are some of the world’s most marginalized people for whom the post-2015 SDGs most matter. If vulnerable migrants, the displaced and other at-risk populations such as victims of human trafficking lack affirmative and repeated identification within the SDG metrics framework, their needs and rights will be ignored in post-2015 development planning initiatives for the next 15 years. In 2015 and 2016, multiple countries introduced regressive measures that undermined access to healthcare for vulnerable non-nationals.

## Recommendations

We have identified four ways to enhance the protection of vulnerable non-nationals’ and internally displaced person’s health and well-being in States’ application of the post-2015 SDG framework. All four recommendations are interdependent and intertwine: not one of the four paths forward that we identify can be effectively achieved without implementation of the other three. Our recommendations are as follows:

- In setting their own post-2015 indicators, UN Member States should explicitly identify and include vulnerable non-nationals, displaced persons and other marginalized groups in the content of such indicators;
- The Inter-Agency and Expert Group on SDG Indicators (IAEG-SDGs) should continue to encourage states and researchers to collect data that will help governments to develop data disaggregation measures and tools that specifically include such populations;
- Statisticians from key multilaterals, together with community and civil society representatives (among other actors), should be actively involved in the formulation and monitoring of SDG indicators at both the global and country-level;
- The inclusion of non-nationals and internally displaced persons in the post-2015 SDG framework must be anchored in the international human right to health.

Our first recommendation is that in setting their own post-2015 indicators, UN Member States from developed and developing nations should explicitly identify and include vulnerable non-nationals, displaced persons and other marginalized groups in the content of such indicators. States should be guided by the overall global goal, while also taking into account their national circumstances [12]. We secondly recommend that the IAEG-SDGs should continue to encourage states, the multilaterals, and researchers to collect data that will help governments to develop data disaggregation measures and tools that specifically include such populations. In addition, national SDG indicators need to incorporate fiscal allocation of resources for emergency and non-emergency level for these specific groups. This is particularly important in the context of SDG 3, which seeks to ensure healthy lives for all, specifically Target 3.7 (“… ensure universal access to sexual and reproductive health-care services…”) and Target 3.8 (“Achieve universal health coverage…”).

Our third recommendation is that statisticians from UNHCR, IOM, and the World Health Organization’s Gender, Equity and Human Rights programme should be actively involved in the formulation of SDG indicators at both the global and country-level. This is likely to involve additional financial and human resource for these international agencies. However, it is well worth the short-term investment. In addition, communities, civil society, foundations and philanthropic organisations, private organizations, and development banks, together with health justice advocates, should also vigorously engage in each country’s formulation of post-2015 indicators. We encourage such actors to reject indicators that do not identify and include the world’s most vulnerable. If country targets and indicators do not best address the “dimensions of inequality that are particularly relevant to each country’s internal borders” [3] - which *includes* the health and related inequalities experienced by vulnerable non-nationals - then we must advocate countries return to the SDG metrics drawing-board.

Finally, we recommend that the inclusion of non-nationals and internally displaced persons explicitly in States’ SDG metrics framework be anchored in the international human right to health, which in turn requires appropriate financing allocations as well as robust monitoring and evaluation processes that can hold technocratic decision-makers accountable for progress. Indeed, it is imperative to acknowledge that all seven billion of us are potentially non-nationals or displaced persons, that we are all human, and that there can be no sustainable development without dignity for all. Human health and planetary survival knows no borders.

## Abbreviations

Go4Health: Goals and Governance for Global Health research consortium
IOM: International Organization for Migration
MDGs: Millennium development goals
SDG: Sustainable Development Goals
UN: United Nations
UNHCR: United Nations High Commissioner for Refugees
WHO: World Health Organization

## References

[1] Brolan CE, Hill PS, Correa-Velez I (2012). Refugees: the Millennium Development Goals’ Overlooked Priority Group. J immigr refug stud.

[2] Rowley EA, Burnham GM, Drabe RM (2006). Protracted refugee situations: parallel health systems and planning for the integration of services. J refug stud.

[3] Hosseinpoor AR, Bergen N, Magar V (2015). Monitoring inequality: an emerging priority for health post-2015. Bull world health organ.

[4] Brolan CE, Dagron S, Forman L, Hammonds R, Abdul Latif L, Waris A (2013). Health rights in the post-2015 development agenda: including non-nationals. Bull world health organ.

[5] United Nations High Commissioner for Refugees (UNHCR) (2015). Mid-Year Trends 2015.

[6] United Nations High Commissioner for Refugees (UNHCR) (2015). World at war. UNHCR Global Trends. Forced displacement in 2014.

[7] International Organization for Migration (IOM), United Nations High Commissioner for Refugees (UNHCR), and partners. Regional Refugee and Migrant Response Plan for Europe Eastern Mediterranean and Western Balkans Route January–December 2016. Available: http://rmrp-europe.unhcr.org/2016_RMRP_Europe.pdf [Accessed 5 February 2016].

[8] International Organization for Migration (IOM) (2015). World migration report 2015. Migrants and cities: New partnerships to manage mobility.

[9] International Organization for Migration (IOM) (2011). World migration report 2010. The future of migration: building capacities for change (executive summary).

[10] Fang ML, Sixsmith J, Lawthom R, Moutian I, Shahrin A (2015). Experiencing ‘pathologized presence and normalized absence’; understanding health related experiences and access to health care among Iraqi and Somali asylum seekers, refugees and persons without legal status. BMC public health.

[11] UN General Assembly Resolution 70/1 (2015). Transforming our world: the 2030 agenda for sustainable development (adopted September 25, 2015).

[12] UN Economic and Social Council. Report of the Inter-Agency and Expert Group on Sustainable Development Goal Indicators (February 19, 2016); E/CN.3/2016/2/Rev.1. Available from: http://unstats.un.org/unsd/statcom/47th-session/documents/2016-2-IAEG-SDGs-E-Revised.pdf [Accessed 28 February 2016].

[13] Biondi P (2016). Human security and external burden-sharing: the European approach to refugee protection between past and present. Int jhum rights.

